# Benefits of Combinations of Vitamin A, C and E Derivatives in the Stability of Cosmetic Formulations 

**DOI:** 10.3390/molecules17022219

**Published:** 2012-02-22

**Authors:** Mirela Donato Gianeti, Lorena Rigo Gaspar, Flávio Bueno de Camargo Júnior, Patrícia Maria Berardo Gonçalves Maia Campos

**Affiliations:** Faculty of Pharmaceutical Sciences of Ribeirao Preto, University of Sao Paulo, Av do Cafe s/n, Monte Alegre, Ribeirao Preto 14040-903, SP, Brazil; Email: mikadg@yahoo.fr (M.D.G.); lorena@fcfrp.usp.br (L.R.G.); flaviobcjr@yahoo.com.br (F.B.C.J.)

**Keywords:** stability studies, cosmetics, vitamins, rheology, shelf-life

## Abstract

Chemically stable ester derivatives of vitamins A, C and E have become a focus of interest for their role in the satisfactory results in skin aging treatments. Accordingly, the aim of this study was to evaluate the physical and chemical stability of a cosmetic formulation containing 1% retinyl palmitate, ascorbyl tetraisopalmitate and tocopheryl acetate, alone or in combination. In the studies of physical stability, a Brookfield rheometer was used to determine rheological behavior of formulations containing the vitamins. Chemical stability was determined by HPLC on a Shimadzu system with UV detection. Results showed that formulations had pseudoplastic behavior and that vitamins did not alter their apparent viscosity and thixotropy. In the chemical stability studies, first-order reaction equations were used for determinations of the shelf-life of vitamins derivatives considering a remaining concentration of 85%. Combined vitamins in a single formulation had a slightly lower degradation rate as compared to different preparations containing only one of the vitamins. Considering that many cosmetic formulations contain vitamin combinations it is suggested that the present study may contribute to the development of more stable formulations containing liposoluble vitamins.

## 1. Introduction

A special interest in vitamins A, C and E has been observed in recent years due to their useful role in the treatment of skin aging and protection of human skin against UV-induced damage [1]. However, esterified vitamin derivatives (retinyl palmitate, ascorbyl tetraisopalmitate and tocopheryl acetate) having improved stability have been preferentially used in topical formulations [2]. 

The greater stability of ester forms of vitamins A, C and E led to studies that demonstrated their effectiveness in cosmetic formulations. Retinyl palmitate acts on the epithelization of dry and rough skin, as well as on the improvement of abnormal keratinization [3]. Ascorbyl tetraisopalmitate, a precursor of Vitamin C reduces cell damage triggered by UVB and effectively suppresses UVB-induced pigmentation [4]. Maia Campos [5] showed a significant effect of a vitamin C derivative in the improvement of skin hydration, which is very important for normal skin metabolism, prevention of skin alterations and early aging. Tocopheryl acetate is a free radical scavenger that may reduce DNA damage and the rate of death in keratinocytes [6]. In addition, it can enhance stratum corneum hydration [7] and reduce skin roughness.

Vitamins may also act synergistically when used in combination [8]. Some studies suggest the importance of combining topical vitamins C and E to protect the skin against UV irradiation and maximize photoprotection [9,10]. Seité [11] showed that topical application of a combination of vitamins A and C is able to reverse, at least in part, skin changes induced by both chronologic and photoaging.

However, vitamin supplemented formulations, even in the ester form, may present low shelf-life, as shown by Guaratini *et al*. [12]. The authors evaluated a gel-cream formulation containing vitamin A palmitate and vitamin E acetate and obtained a shelf-life of only 77 days (at 45, 37 and 25 °C and 75% relative humidity), characterized by a first-order degradation reaction of the vitamin A derivative. 

Beyond the characteristics of the active substance, physical and chemical properties of the vehicle such as pH and water content and the combination of active substances should be considered to assure the stability of cosmetic formulations containing vitamins. The vehicle raw materials in cosmetic formulations must be compatible with the active substances selected to fulfill the indicated use of the product [13].

Physical and chemical stability of cosmetic formulations containing vitamins A, C and E derivatives must be determined to evaluate quality, since physical characteristics of the formulation and vitamin integrity must be kept constant during the formulation shelf-life. 

The physical stability of a cosmetic formulation can be evaluated by studying rheological behavior under thermal stress. In this condition, prediction of instability processes may be possible since some parameters such as viscosity, solubility, creaming facilitation, coalescence, melting of waxes or hydration of polymers are altered [12]. Therefore, it is important to understand the rheological behavior when formulating, handling, mixing, processing, transporting, and storing such formulations [14]. Furthermore, the performance of dermatological products depends to a great extent on rheological behavior.

HPLC measurements are currently used to obtain chemical stability data about formulations over time [14]. To predict formulation shelf-life the kinetics of chemical degradation may be mathematically treated by using the Arrhenius equation [15]; however physical stability is not considered in this case. Prediction based only on chemical parameters cannot be totally trusted especially for cosmetic formulations where physical stability is of utmost importance and other parameters should also be analyzed.

Clinical studies performed by our research group showed the importance of cosmetic formulations containing combinations of liposoluble derivatives of vitamins A, C and E in the improvement of skin conditions [16]. Considering that there are no studies that evaluate the stability of a cosmetic formulation containing these liposoluble derivatives in combination, it is very important to understand the influence of the vehicle on the degradation rate of the components as well as on the shelf-life of the formulation.

The aim of this study was to evaluate the physical and chemical stability of a cosmetic formulation containing 1% of retinyl palmitate, ascorbyl tetraisopalmitate and tocopheryl acetate, alone or in combination. 

The results obtained should contribute to a better understanding of the stability of vitamin derivatives added to cosmetic formulations and of the physical and chemical stability of the proper formulations and to predict their shelf-life as well.

## 2. Results and Discussion

### 2.1. Physical Stability

Rheological properties are the most important parameters to be measured in the development of a topical formulation. They characterize features of spreadability, *i.e.*, the consumer's first contact with the formulation. In addition the rheological characteristics may also be an indication of changes caused by the addition of some active substances [17].

**Figure 1:**
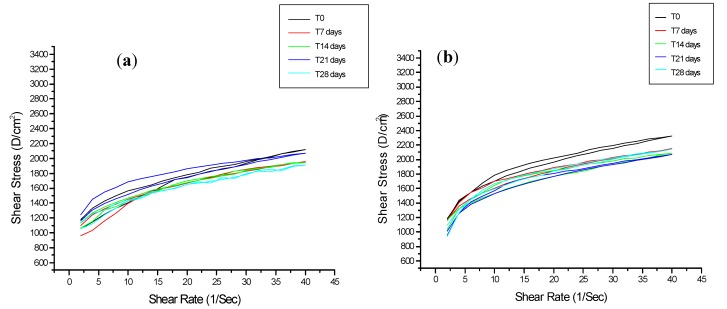
Rheograms of formulations supplemented (**b**) or not (**a**) with the combination of vitamins under study, when stored at 25 °C, during 0, 7, 14, 21 and 28 days after preparation. (**a**) formulation with vitamins in combination stored at 25 °C; (**b**) formulation without vitamins stored at 25 °C.

**Figure 2:**
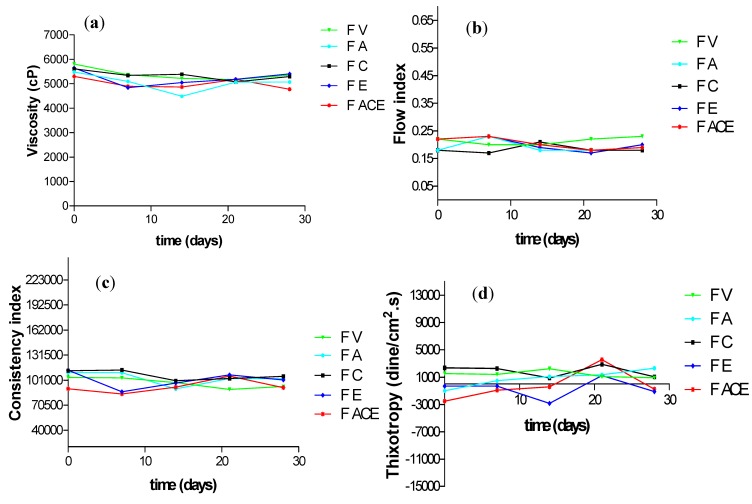
Alterations in the apparent viscosity (**a**), flow index (**b**), consistency index (**c**) and thixotropy (**d**) of vehicle (F V), or the vehicle supplemented with vitamin A (FA), vitamin C (FC), vitamin E (FE) or with the combination of these vitamins (F ACE) initially (0), 7, 14, 21 and 28 days after preparation, at room temperature.

In this study, rheological parameters indicated that addition of the vitamins derivatives under study to the vehicle formulation did not compromise its structure, since no peaks or other alterations in the rheogram curves were observed (Figure 1). Data on the apparent viscosity, flow index, consistency index and thixotropy during the experimental period is shown in Figure 2.

It indicates that the addition of vitamins derivatives did not produce any alterations in the apparent viscosity or on the consistency index of the formulations. Furthermore, no alterations were detected by storage at 37 and 45 °C (Figure 2A,C). 

The results showed that the formulations had pseudoplastic behavior, with a flow index below 1, which was not altered by the addition of vitamins to the vehicle formulation (Figure 2B). Pseudoplasticity is a desirable rheological property in cosmetic formulations. It improves application and spreading providing a pleasant sensory feeling. Newtonian materials do not behave in this way, because when spread on the skin they run very quickly [18].

The consistency index of the formulations during the study was not changed by the addition of the vitamin derivatives. Similarly, hysteresis areas (thixotropy), a pseudoplastic natural characteristic observed in the rheograms (Figure 1), were not changed by vitamins (Figure 2D). 

Antithixotropic behavior (negative thixotropy values) observed during the experiment could be due to the raw material employed and does not indicate instability since some authors suggest that high molecular weight polymer solutions present anomalous behavior under shear flow [19].

The present study showed that, over time, physical alterations are not prominent in formulations with added single vitamins or combined (Figure 2). 

### 2.2. Chemical Stability

HPLC with UV detection was the method chosen to verify chemical stability of formulations included in the study. The method, a common procedure used in the analysis of drugs and cosmetics [12] allows quantitative and qualitative assessment of vitamins. Vitamin K1 was the internal standard; the compound has chemical stability and fat-soluble characteristics like vitamins A, C and E [12,20].

**Figure 3:**
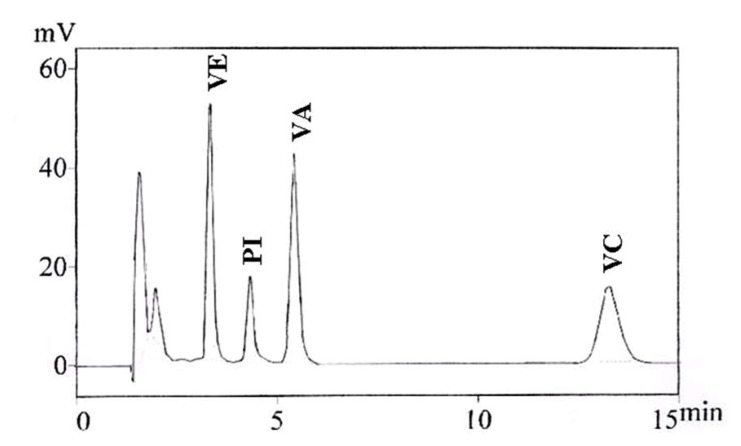
HPLC chromatograms of vitamin E (VE), A (VA) and C (VC) derivatives extracted from the gel-cream formulation in concentration of 250 μg/mL, with Vitamin K1 (PI) as an internal standard.

A representative HPLC chromatogram of vitamin extracts from gel-cream formulations is depicted in Figure 3. It shows well resolved Vitamins E, K1, A and C peaks with retention times of about 3.0, 4.3, 5.5 and 12.3 min, respectively, in a single and short easily reproduced analysis. 

The precision and accuracy/recovery of the method (intra- and inter-assay) are presented in Table 1. Considering the involvement of procedures as extraction, filtration and dilution the precision and accuracy of the analytical method were acceptable with intra/inter assay values of 1.34 to 2.54 and of 97.26 to 111.07, respectively.

**Table 1:** Intra- and inter-days precision and accuracy/recovery (%) in the quantification of vitamins A, C and E extracted from experimental formulations.

Vitamin derivative	Theoretical concentration (μg·mL^−1^)	Intra-day			Inter-day		
		Obtained concentration ± S.D.^a^ (μg mL^−1^)	Precision (R.S.D. %)	Accuracy/ recovery (%)	Obtained concentration ± S.D.^b^ (μg·mL^−1^)	Precision (R.S.D. %)	Accuracy/ recovery (%)
A	250	243.13 ± 3.26	1.34	97.26	243.43 ± 3.26	1.34	97.37
C	250	277.68 ± 7.05	2.54	111.07	276.00 ± 6.68	2.42	110.40
E	250	276.34 ± 5.36	1.94	110.54	275.23 ± 5.68	2.06	110.09

^a^ Standard deviation (intra day n = 6); ^b^ Standard deviation (inter day n = 18).

The values of the vitamin degradation constant, K, were almost the same, indicating that the reaction of degradation followed a first-order reaction, which is in agreement with previous reports [12,21,22] on retinyl palmitate degradation in emulsions. Consequently, shelf-life of vitamins A, C and E were calculated using 1st order chemical kinetics equations and 85% remaining concentration.

**Figure 4:**
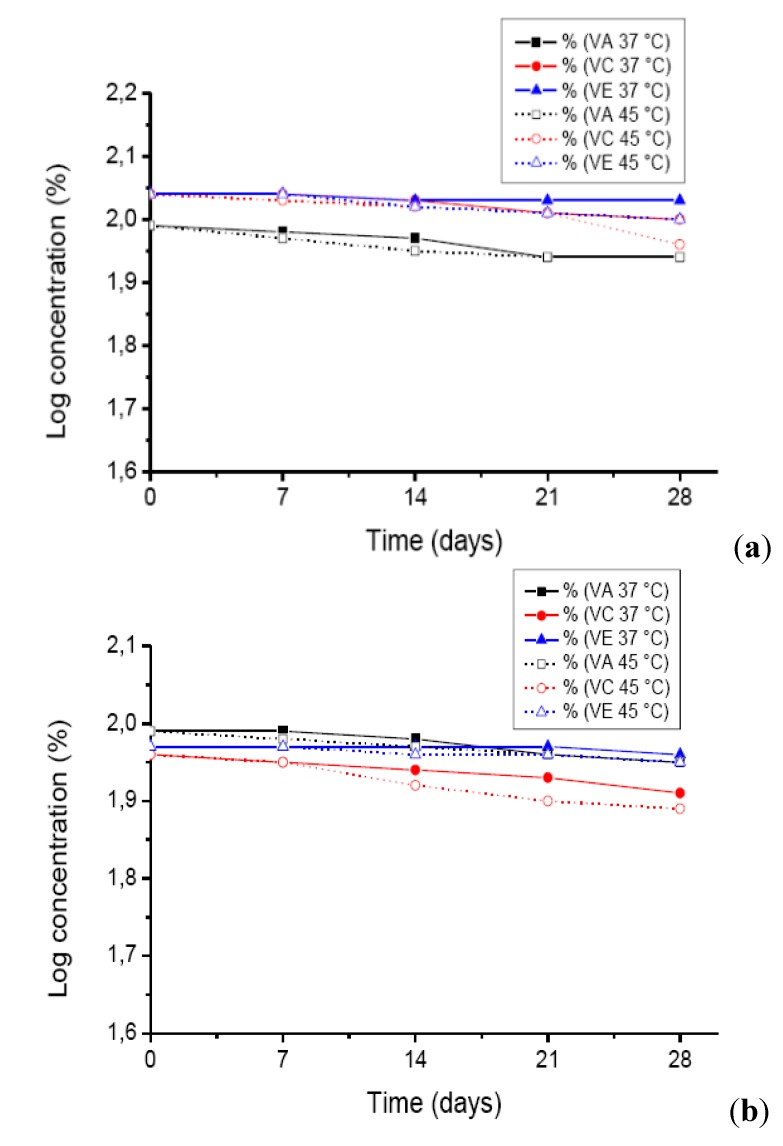
Quantification of vitamin A palmitate (VA), vitamin C (ascorbic acid (VC)) and vitamin E acetate (VE), expressed as logs of concentration values over time, when added, alone (**a**) or in combination (**b**) to the formulation under study stored at 37 and 45 °C with 75% RH.

Formulations containing vitamins, alone or in combination, presented different degradation kinetics for vitamins A, C and E (Figure 4A and 4B). When these vitamins were combined in one formulation, their degradation rate was slightly lower than when they were alone, in different formulations. The shelf-life of vitamins contained in combination was 120, 318 and 1,116 days for vitamin A, C and E, respectively (Table 2). For single vitamins the values were 61, 173 and 757 for vitamin A, C and E, respectively (Table 2). The same results were obtained experimentally in formulations maintained at room temperature, which validates the accelerated method employed for chemical stability determinations.

**Table 2:** Shelf-life of vitamins derivatives A, C and E, when added, alone (in different formulations) or in combination (in the same formulation), stored at 45 and 37 °C, 75% RH.

Vitamins derivatives	Shelf-life (days)	
	Alone	in combination
Retinyl palmitate	61	120
Ascorbyl tetraisopalmitate	173	318
Tocopheryl acetate	757	1116

Studies of accelerated stability in the cosmetic area have been performed in periods of 30 to 90 days, but it is suggested that the study of chemical stability and the shelf-life of a cosmetic formulation should be conducted for a minimum period of three months at the temperatures of 37 and 45 °C, in the same way as performed for pharmaceutical products [23]. 

Shelf-life of gel-cream formulations with silicones containing combinations of vitamin A palmitate and vitamin E acetate were shown to be very low and that the addition of the antioxidant DL-alpha-tocopherol enhanced the periods to 77 days [12]. Other authors observed that the addition of antioxidants such as BHT, Ronoxan A®, alpha-tocopherol and glutathione increased the shelf-life of formulations containing vitamin A palmitate [24]. 

In this study the shelf-life for vitamin A palmitate was higher than the one obtained by Guaratini *et al*. [12], even when the gel-cream studied was supplemented with DL-alpha-tocopherol. This was probably due to differences between the two formulations under study, since the one used by Guaratini *et al*. [12] had high levels of water, which enhances hydrolysis reactions [19]. The formulation developed in this study contains ethylhexyl ethylhexanoate, which is a high molecular weight emollient that can reduce the electrical conductivity of the medium, and consequently the hydrolysis of retinyl palmitate. 

Battna *et al*. [25] showed that combinations of vitamin A palmitate and vitamin E acetate caused a significant reduction of fat oxidation. Our results are similar to the ones obtained by Battna *et al*., since when these vitamins were combined in the same formulation, all of them presented slightly lower degradation constants, K.

Thus, the results obtained in this study suggest a positive interaction between the liposoluble vitamins A, C and E, besides the synergism in efficacy studies reported in the literature [9,10,11]. Combined vitamin derivatives did not alter the physical characteristics of the vehicle, as observed in the rheological study; but enhanced the stability of the formulation by increasing the shelf life of each component. Thus, these results are consistent with the ones that observed a better stability of vitamins in combination [12,25,26]. 

The reasons for the improvement of the formulation stability by the combination of liposoluble vitamins A, C and E are not entirely clear; however, it is suggested that an intermolecular interaction of vitamins derivatives may reduce the reactivity of molecular oxygen, thus increasing their own stability in the formulation. 

Another hypothesis is that, the three times higher concentrations of active substances in the formulation of combined vitamins compared to single additions, provides a stronger competition for the oxygen molecules thus reducing free oxygen reacting with other formulation raw materials. 

These results are similar to the ones obtained by Darvin *et al*. [7] who observed that an optimal mixture of antioxidants protected the formulations against oxidation, extending their working life and the protection of the skin against the negative action of produced free radicals. Benevenuto *et al*. [27] also showed that formulations containing vitamin A palmitate with UV-filters combined with some photostabilizers had better protective effects on hairless mice skin than the ones without any photostabilizer. Considering that many cosmetic formulations contain combinations of vitamins, the results in the present study could also contribute to the development of more stable formulations.

## 3. Experimental

### 3.1. Formulations

Formulations based on acrylate crosspolymer and methylphenyl polysiloxane (Table 3), were prepared in a Heidolph RZR 2021(Schwabach, Germany) stirrer at 650 rpm and supplemented or not with 1.0% retinyl palmitate (1,700,000 UI/g), 1.0% of tocopheryl acetate (DSM Nutritional Products, Basel, Switzerland), 1.0% of ascorbyl tetraisopalmitate (Nikko Chemicals, Tokyo, Japan), or a combination of these vitamin derivatives in the same concentration. 

**Table 3:** Components of the formulation under study.

Components	Percentage of components in the formulation (w/w)
Acrylate crosspolymer	1.00
Methylphenyl Polysiloxane	4.00
Glycerin 86%	3.00
Ethylhexyl Ethylhexanoate	3.00
BHT	0.05
Propyleneglycol	3.00
Phenoxyethanol and parabens	0.80
Distilled water	85.15

### 3.2. Stability Studies

Formulations containing vitamins A, C and E, were stored in PVC pots (37 mm in diameter × 29 mm deep), at 45, 37 and 25 °C and 75% relative humidity (RH) for up to 189 days. Rheological measurements and sample collection for vitamin quantification were made at 7-day intervals during 28 days. Vitamin quantifications were also performed after 63, 126 and 189 days of storage. All vitamin quantifications were made by a validated isocratic HPLC method. To predict formulation shelf-life its results were mathematically treated by using the Arrhenius equation, which characterizes formulations chemical stability [28,29]. Considering that an increase in storage temperature can decrease the chemical stability of a formulation [15], vitamin quantifications also represented degradation kinetics. Thus, chemical stability was calculated as the period of time in which formulations maintained at room temperature (25 °C), show a maximum of 15% loss in the concentration of their main components.

### 3.3. Physical Stability

Physical stability was assessed by rheological determinations performed in a model DV-III Brookfield rotational rheometer (Stoughton, MA, USA) with a cone-plate configuration, connected to a Brookfield software program, RHEOCALC Version V 1.01. Rheograms and viscosity measurements were made under the following experimental conditions: 25 °C, 0.5 g samples and CP52 spindle. To obtain the ascendant curve, rotation speeds were progressively higher (1 to 20 rpm) and the procedure was repeated in reverse with gradually decreasing speeds (20 to 1 rpm) for the descendant segment. The rheograms obtained were mathematically analyzed by the Ostwald Law, where values of apparent viscosity, flow index (related to the degree of sample pseudoplasticity) and consistency index were obtained. The numeric integration of the rheogram curves was made by the Software MICROCAL ORIGIN and the area under the ascendant and descendant curves was obtained (loop area hysteresis-thixotropy) [18].

### 3.4. Chemical Stability

The HPLC system consisted of a model LC-10AD Shimadzu Liquid Chromatograph (Kyoto, Japan) fitted with a variable wavelength UV detector (SPD-10A), connected to a personal computer. The integrator program consisted of a LC-10, Shimadzu. Chromatographic separations were performed on a LiChrocart (125, 4 mm) Merck column, filled with 5 µm Lichrospher 100 RP-18 particles as the stationary phase. Degassed methanol and *iso*-propanol (45:55 w/w) was the mobile phase at a flow rate of 0.8 mL·min^−1^. The injection volume was 20 µL and analyses were performed at 235 nm. Vitamin K1 (phytonadione, Sigma, St. Louis, MO, USA) was the analyzed internal standard; formulation samples (25 mg) were added to a 25 mL glass beaker containing 8 mL of iso-propanol and dissolved ultrasonically [12,30]. Then, the solution was filtered and used for HPLC injection. To quantify retinyl palmitate, tocopheryl acetate and ascorbic acid derivatives in the formulations, standard solutions were prepared daily and analyzed by HPLC in parallel to samples. Peak-area ratios were used for calculations following the internal standard method. For precision assays, samples were analyzed six times and the intra-assay relative standard deviation (R.S.D.) calculated. Inter-assay R.S.D. was determined analyzing the samples six times in three different days. The intra and inter-assay accuracy/recovery was determined by a comparison between the theoretical concentrations of standard substances added to topical formulations analyte-free and those obtained from the chromatographic analysis [12,30].

## 4. Conclusions

Stability studies indicate that formulations studied showed acceptable stability mainly when physical aspects were considered, which are very important in the development of topical formulations. 

Formulations containing vitamin derivatives in combination showed better chemical stability. Considering that many cosmetic formulations contain vitamins in combination to improve their efficacy, our results suggest that the formulation stability is also enhanced under these conditions.

Finally, it is concluded that the results in this study contribute to the development of more stable formulations containing liposoluble vitamins derivatives in combination. In addition, the results contribute to the understanding of cosmetic physical and chemical stability by using simple and rapid analyses to predict the shelf-life of these products.
